# Obesity-Associated Colorectal Cancer

**DOI:** 10.3390/ijms25168836

**Published:** 2024-08-14

**Authors:** Lucia Gonzalez-Gutierrez, Omar Motiño, Daniel Barriuso, Juan de la Puente-Aldea, Lucia Alvarez-Frutos, Guido Kroemer, Roberto Palacios-Ramirez, Laura Senovilla

**Affiliations:** 1 Unidad de Excelencia Instituto de Biología y Genética Molecular (IBGM), Universidad de Valladolid–CSIC, 47003 Valladolid, Spain; lucia.gonzalezg@uva.es (L.G.-G.); omar.motino@uva.es (O.M.); daniel.barriuso@uva.es (D.B.); jpualdea@gmail.com (J.d.l.P.-A.); lucia.alvarez.frutos@uva.es (L.A.-F.); roberto.palacios@uva.es (R.P.-R.); 2 Centre de Recherche des Cordeliers, Equipe Labellisée par la Ligue Contre le Cancer, Université Paris Cité, Sorbonne Université, Inserm U1138, Institut Universitaire de France, 75006 Paris, France; kroemer@orange.fr; 3 Metabolomics and Cell Biology Platforms, Institut Gustave Roussy, 94805 Villejuif, France; 4 Institut du Cancer Paris CARPEM, Department of Biology, Hôpital Européen Georges Pompidou, AP-HP, 75015 Paris, France

**Keywords:** colorectal cancer, obesity, epidemiology, risk factors, molecular factors, treatments, targets

## Abstract

Colorectal cancer (CRC) affects approximately 2 million people worldwide. Obesity is the major risk factor for CRC. In addition, obesity contributes to a chronic inflammatory stage that enhances tumor progression through the secretion of proinflammatory cytokines. In addition to an increased inflammatory response, obesity-associated cancer presents accrued molecular factors related to cancer characteristics, such as genome instability, sustained cell proliferation, telomere dysfunctions, angiogenesis, and microbial alteration, among others. Despite the evidence accumulated over the last few years, the treatments for obesity-associated CRC do not differ from the CRC treatments in normal-weight individuals. In this review, we summarize the current knowledge on obesity-associated cancer, including its epidemiology, risk factors, molecular factors, and current treatments. Finally, we enumerate possible new therapeutic targets that may improve the conditions of obese CRC patients. Obesity is key for the development of CRC, and treatments resulting in the reversal of obesity should be considered as a strategy for improving antineoplastic CRC therapies.

## 1. Introduction

Colorectal cancer (CRC) affects approximately 2 million people and is the second leading cause of mortality worldwide, accounting for 9.3% (~900,000 deaths). Although the incidence of CRC has increased by 70% since the 1990s, the 5-year mortality and prevalence rates have decreased because of early detection, prevention, and new therapies. Currently, the 5-year and 10-year survival rates are 65% and 58%, respectively. However, the International Agency for Research on Cancer (IARC) projects a 56% increase in CRC by 2040 [1,2]. Certain factors, such as age, genetics, and lifestyle, play a key role in CRC. The incidence and mortality rates are higher in men than in women, with the highest rates in non-Hispanic African Americans [1,3]. Recent data show that the incidence of CRC is increasing in adults younger than 50 years (early-onset colorectal cancer, EOCRC). This trend is in part associated with the Western diet, which directly affects the composition of the gut microbiota and indirectly affects the body metabolism [4,5,6]. In fact, epidemiologic studies show that overweight and obesity are responsible for 11% of CRC cases [7].

According to the World Health Organization (WHO), by 2022, 2.5 billion adults were overweight and 890 million were obese. Overweight and obesity are defined as an abnormal or excessive accumulation of fat, and obesity is now considered as a chronic disease risk. A body mass index (BMI) greater than 25 kg/m^2^ is considered overweight, and greater than 30 kg/m^2^ is considered obese [8]. The Global Burden of Diseases, Injuries, and Risk Factors (GBD) 2019 study has established a hierarchy of risk factors classified as levels 1 to 4 with a total of 87 risks or risk groups, including environmental, lifestyle, and metabolic risks. The lifestyle risks include dietary risks, such as diets low in fruits, vegetables, legumes, whole grains, nuts and seeds, milk, fiber, calcium, marine-derived omega-3 (ω3) fatty acids, and polyunsaturated fatty acids, as well as diets high in red meat, processed meat, sugary beverages, trans-fats, and sodium. The metabolic risks include high fasting plasma glucose, high low-density lipoprotein (LDL) cholesterol, high systolic blood pressure, high BMI, low bone mineral density, and renal dysfunction [9]. BMI, waist circumference, and increased waist-to-hip ratio are associated with significant increases in CRC in men. However, this association appears to be less strong in women, probably because of the protective effect of estrogens. In addition, the data suggest that weight loss, by diet or bariatric surgery, is associated with a lower incidence of CRC [7,10,11,12,13,14].

In this review, we examine the latest data on the epidemiology, risk factors, molecular factors, current treatments, and emerging targets for obesity-associated CRC.

## 2. Obesity-Associated Colorectal Cancer Epidemiology

CRC encompasses cancers of the colon and rectum. Clinical staging is employed to inform therapeutic decision-making. The earliest stage is stage 0, also known as carcinoma in situ, which is defined by the presence of malignant cells in the colon or rectum mucosa, which have not yet reached the muscularis mucosae layer. In stage I, the disease progresses with the invasion of proliferating tumor cells into the submucosal or muscularis propria layers but without reaching the adjacent organs or lymph nodes. Stage II CRC has advanced to the outer layers of the rectum and colon but has not yet spread to the lymph nodes or other organs. In stage III, the tumor cells invade the lymph nodes. Finally, stage IV is defined as the spread of the CRC tumor cells to at least one distant organ. The most common sites of metastasis for CRC are the liver, lung, and bone. These metastases typically occur between 0 and 5 years after progression in the absence of successful treatment [15]. The sigmoid colon is the most common site for CRC, with an incidence of 55%. Other common sites include the ascending colon (23.3%), transverse colon (8.5%), and the descending colon and cecum, with an incidence of approximately 8% each [16]. A worse prognosis is associated with tumors occurring in the ascending section of the colon, especially in women. Indeed, 26% of CRC diagnoses concern this area [17]. It is estimated that 80 to 85% of CRC cases are spontaneous, arising from the formation of colorectal adenomatous polyps (also known as colorectal adenoma, CRA) that progress to carcinomas. Only 15–20% of CRC patients exhibit a family history, and 5% of patients suspicious of a hereditary syndrome are eligible for detection through germline testing [18]. Histological analysis indicates that the most prevalent pathology is CRA (90–95% of patients), followed by mucinous adenocarcinoma (3.95%). Other histotypes, such as epidermoid carcinomas, carcinoids, sarcomas, melanomas, and atypical CRC lymphomas, are less common (1.05–5.05% for the other types) [18].

Visceral adiposity, along with its aggravation, is associated with CRA [19,20,21,22]. A higher BMI is associated with an increased risk of developing CRA [23,24]. Moreover, individuals with obesity but without obesity-related metabolic abnormalities have a higher prevalence of CRA [25].

CRCs can result from inherited germline mutations (transmitted by the patient’s parents) or—more frequently—from sporadic mutations in the tissue that develops into the tumor. Approximately 80% of CRCs are considered sporadic [26]. The presence of one or two affected family members with cancer increases the likelihood of cancer, which translates to a 15–20% increase in cancer risk [27,28]. The most prevalent familial syndrome is hereditary nonpolyposis CRC, or Lynch syndrome, which accounts for 2–5% of all cases of familial CRC. Lynch syndrome is caused by mutations in DNA mismatch repair (MMR) genes, including MutS homolog 2 (*MSH2*; 38% of cases), MutL protein homolog 1 (*MLH1*; 59% of cases), and, to a lesser extent, *MSH6* and PMS1 homolog 2, mismatch repair system component (*PMS2*) [29]. It has been demonstrated that obesity is associated with an increased risk of CRC in patients with Lynch syndrome [30]. Specifically, obese men with Lynch syndrome have a twofold increased risk of CRC compared to non-obese men. Specifically, obese subjects with an *MLH1* mutation have a 49% increased risk of CRC [31]. Familial adenomatous polyposis (FAP), a condition in which patients develop hundreds of adenomatous polyps, accounts for less than 1% of all CRC. This hereditary syndrome is caused by a germline mutation in the adenomatous polyposis coli (*APC*) gene. Another hereditary syndrome is the polyposis associated with a mutation in the *MUTYH* gene (MUTYH-Associated Polyposis, MAP), which typically occurs in young adults (40–60 years) [32]. Peutz–Jeghers syndrome is caused by mutations in serine/threonine kinase 11 (*STK11*) (also known as *LKB1*) [33]. To our knowledge, obesity has not yet been associated with the risk of developing CRC in FAP or Peutz–Jeghers syndrome.

**Figure 1:**
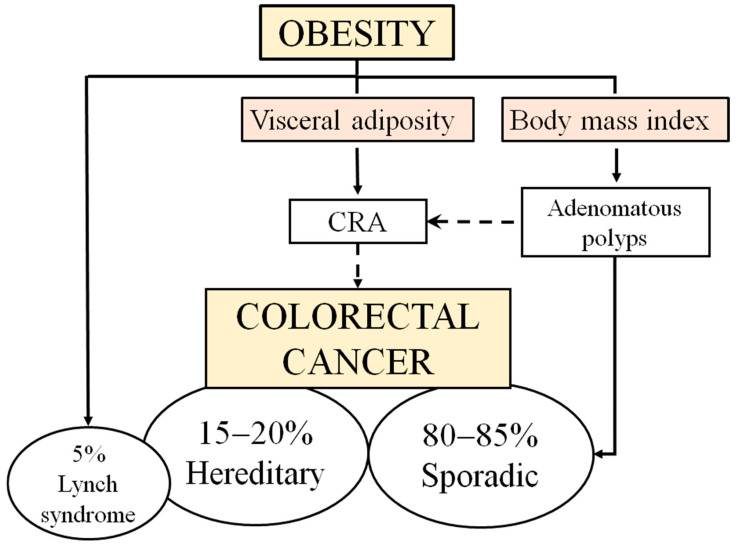
The epidemiology of colorectal cancer (CRC) associated with obesity. Obesity is associated with an increased risk of developing CRC. A positive correlation has been identified between body mass index and the presence of adenomatous polyps, which may develop into CRC. It has been demonstrated that visceral adiposity is associated with colorectal adenoma (CRA), the precursor of CRC. CRC can be sporadic or hereditary. Lynch syndrome is a form of hereditary cancer. Obesity increases the risk of CRC in patients with Lynch syndrome.

Given the considerable heterogeneity of CRC, an international consortium was established almost 10 years ago with the aim of identifying the distinct subtypes of this tumor to enhance patient stratification and to establish therapeutic protocols tailored to each subtype. The consensus molecular subtype (CMS) classification of CRC comprises four major groups: CMS1 (immune), CMS2 (canonical), CMS3 (metabolic), and CMS4 (mesenchymal). The CMS1 (14%) subtype is characterized by high microsatellite instability (MSI) and signs of an ongoing immune response. The CMS2 subtype (37%) exhibits the highest chromosomal instability (CIN) and is typically associated with epithelial cell differentiation. Furthermore, it exhibits the activation of several classical signals, including the *WNT* and *MYC* oncogenes, both of which play a role in the metabolic reprogramming of cancer cells, affecting processes such as glycolysis, glutaminolysis, and lipogenesis. Tumors of the CMS3 subtype (13%) are associated with metabolic dysregulation and epithelial features. The subtype with the poorest prognosis, CMS4 (23%), is associated with epithelial–mesenchymal transition (EMT) and strong transforming growth factor β (TGF-β) signaling, stromal invasion, and angiogenesis [34]. A transcriptomic analysis of the CRC samples from obese patients revealed that tumor EMT is a significant factor in the relationship between obesity and colon cancer. Obesity has been shown to enhance the immune phenotypes of CMS1, CMS2, and CMS4 tumors, which is consistent with the fact that obesity is associated with systemic inflammation. Furthermore, there is evidence that obesity may enhance the mesenchymal characteristics of the CMS4 subtype and induce a metabolic phenotype associated with CMS3 tumors [35]. The cancer cells of the CMS4 subtype reprogram lipid metabolism due to the activation of the nuclear factor kappa-light-chain-enhancer of activated B cells (NFκB) pathway, via triacylglycerol lipase (TAG) carboxylesterase 1 (CES1), thus establishing a connection between obesity-associated inflammation and metabolic adaptation [36]. Of note, obese patients have a worse prognosis across all the CMS subtypes [35] (Figure 1).

Physical activity reduces the relative risk of CRC by 21–27% [37]. A US study of patients of different ethnicities concluded that overweight or obese patients who exercised one or more hours per week had a lower prevalence of CRC [38]. A Korean study found that increased physical activity was associated with a lower prevalence of CRA (odds ratio (OR) = 0.56; 95% confidence interval (CI) 0.40–0.79) [39]. Furthermore, a prospective study in the same country found that participation in vigorous physical activity [hazard ratio (HR), 0.84; 95% CI, 0.72–0.97] and walking (HR, 0.84; 95% CI, 0.72–0.98) were associated with a lower overall risk of cancer, including a lower risk of CRC (HR, 0.46; 95% CI, 1.06–1.40), in men. In addition, the analysis showed that the risk of CRC was low among the men in the overweight group who participated in climbing activities (HR, 0.61; 95% CI, 0.37–1.00) [40]. A Scandinavian study also associated physical activity with a lower risk of colon cancer (9% [2–16%]), and, when high physical activity and low BMI were combined, the relative risk reduction for colon cancer was 27% (19–35%) [41]. The prospective Melbourne Collaborative Cohort Study found that patients who exercised had longer survival from CRC (HR, 0.73; 95% CI, 0.54–1.00), especially for stages II–III tumors (HR, 0.49; 95% CI, 0.30–0.79]). An increasing body fat percentage leads to increased CRC deaths (HR, 1.33 per 10 kg; 95% CI, 1.04–1.71), just as increasing waist circumference decreases CRC survival (HR, 1.20 per 10 cm; 95% CI, 1.05–1.37) [42].

Healthy dietary patterns characterized by high intakes of fruits and vegetables, cereals, and lean meat/fish and low intakes of red meat/processed meat and refined carbohydrates have been associated with a lower risk of CRC. Habits such as fish consumption can reduce the risk of CRC by 12%, intake of more than 20 g/day of dietary fiber by 25%, and consumption of 525 mL/day of milk by 26% in men [43]. Several prospective studies show an inverse association between healthy diet indices and CRC risk. For example, the highest score on the Healthy Eating Index (HEI) is inversely associated with CRC risk in both healthy-weight and overweight/obese participants [44], as is the Dietary Antioxidant Index (DAI) with respect to CRC risk in overweight/obese individuals [45], and the Italian Mediterranean Index (IMI) with respect to CRC risk [46].

In fact, the European Prospective Investigation into Cancer and Nutrition study has shown that following a Mediterranean diet reduces the risk of CRC in both healthy-weight individuals and obese men [47,48].

## 3. Risk Factors for Obesity-Associated Colorectal Cancer

Despite a decline in CRC-related mortality among individuals aged 50 and above due to screening programs resulting in earlier diagnosis, the CRC-related mortality has risen among individuals under the age of 50, in particular in high-income countries. This increase in CRC cases and deaths in younger individuals is attributable to dietary and other lifestyle-related risk factors [49].

The non-modifiable risk factors include age, gender, ethnicity, genetic predisposition, family history of CRC, abdominopelvic radiation, gut microbiota, and comorbidities such as inflammatory bowel disease (IBD), ulcerative colitis and Crohn’s disease, cystic fibrosis, renal transplantation, coronary heart disease, bacterial and viral infections, antibiotic use, diabetes mellitus, and insulin resistance [18]. There is substantial evidence indicating that men are more susceptible to CRC than women, with a 4:1 ratio in terms of incidence. The ethnic group with the highest incidence and mortality is non-Hispanic African Americans [50,51,52]. The multifactorial studies on comorbidities have demonstrated that age and prior diseases increase the risk of CRC. In particular, IBDs, including ulcerative colitis and Crohn’s disease [53,54], are associated with approximately 2% of CRC deaths and a poor 5-year survival rate of 50% [55].

**Figure 2:**
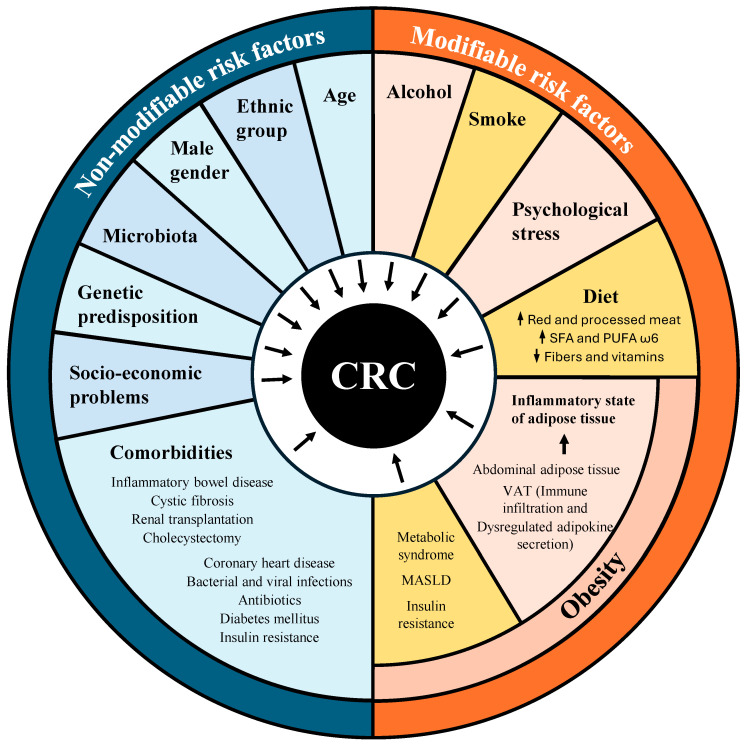
Risk factors contributing to the development of obesity-associated colorectal cancer (CRC). Obesity, diet, and comorbidities play a crucial role in the development of CRC. MASLD, metabolic dysfunction-associated steatotic liver disease; PUFA, polyunsaturated fatty acids; SFA, long-chain saturated fatty acids; VAT, visceral adipose tissue; ↑, high; ↓, low.

It is noteworthy that excess body fat at a young age may contribute to the EOCRC [56,57], but even obesity developing at the adult stage predisposes to CRC [58]. Other modifiable risk factors for CRC include alcohol consumption, smoking, obesity, a sedentary lifestyle, an unhealthy diet, and psychological stress [18] (Figure 2).

The overall level of body fat, as determined by BMI measurement, shows a consistent correlation with the risk of developing CRC. This correlation is more pronounced in men than in women. However, abdominal obesity (as determined by the waist circumference or waist-to-hip ratio) is a risk factor for colon cancer in both sexes. Thus, a preponderantly visceral fat distribution is a more significant CRC risk factor than body weight or BMI [59] regardless of the presence of CRA [21,22]. Visceral adipose tissue (VAT) secretes adipokines that are involved in inflammation, coagulation, and other metabolic actions. Abdominal obesity is associated with insulin resistance, dyslipidemia, and systemic inflammation, all of which are implicated in CRC [59].

**Figure 3:**
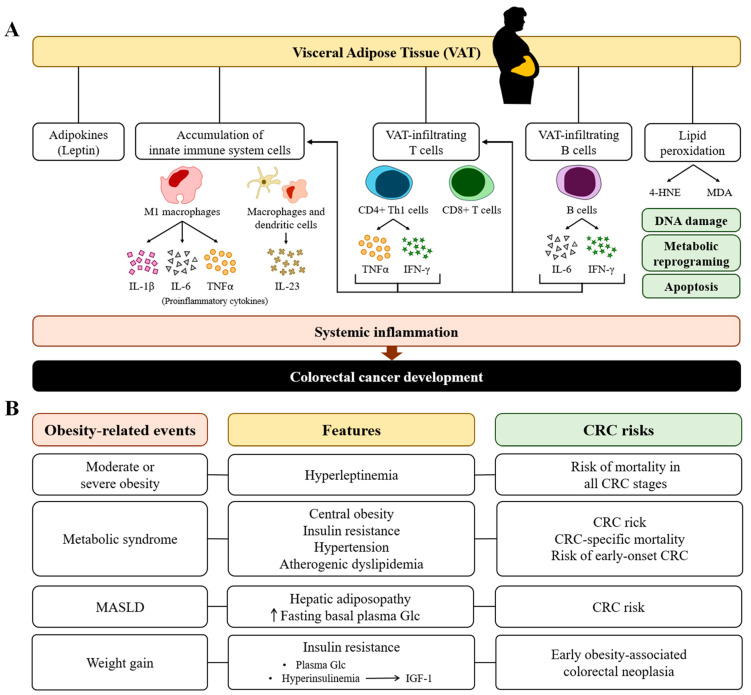
Molecular and cellular factors that are key players involved in the relationship between obesity and colorectal cancer (CRC) development. (**A**) Relationship of visceral adipose tissue (VAT) to systemic inflammation as a driver of CRC development. VAT leads to the production of adipokines; accumulation of innate immune cells, such as M1-polarized macrophages secreting IL-1β, IL-6, and TNF-α, and IL-23-secreting macrophages and dendritic cells; T-cell infiltration, such as CD4+ Th1 T cells secreting TNF-α and IFN-γ and CD8+ T cells; B-cell infiltration secreting IL-6 and IFN-γ; and lipid peroxidation leading to 4-HNE and MDA production, causing DNA damage, metabolic reprogramming, and apoptosis. All these events induce systemic inflammation that favors CRC development. (**B**) Characteristics of obesity-related events associated with CRC risk. Obesity-related events such as moderate or severe obesity, metabolic syndrome, MASLD, and weight gain lead to an increased risk of CRC through various mechanisms, such as hyperleptinemia, insulin resistance, hypertension, atherogenic dyslipidemia, hepatic adiposopathy, or increased basal Glc in fasting blood. 4-HNE: 4-hydroxy-2-nonenal; Glc: glucose; IGF-1: insulin-like growth factor 1; IL: interleukin; IFN-γ: interferon γ; MASLD: metabolic dysfunction-associated steatotic liver disease; MDA: malondialdehyde; TNF-α: tumor necrotic factor α; VAT: visceral adipose tissue; ↑, elevated.

Since 2011, tumor-promoting inflammation has been identified as one of the hallmarks of cancer [60]. The generalized inflammatory state of adipose tissues is a crucial factor in the association between obesity and CRC because VAT is a major source of proinflammatory metabolites and cytokines [61] (Figure 2). The obesity-related increase in VAT is characterized by the accumulation of macrophages, which are predominantly polarized M1 state and hence produce proinflammatory cytokines, including interleukin (IL)-1β, IL-6, and tumor necrosis factor-α (TNF-α). Additionally, lymphocytes infiltrate adipose tissues in obesity. These include CD8+ cytotoxic T cells and CD4+ Th1 cells that secrete TNF-α and interferon (IFN)-γ, which favor the M1 polarization of macrophages. VAT-infiltrating B cells secrete proinflammatory cytokines such as IL-6 and IFN-γ, thereby favoring the activation of T cells and macrophages. This immune infiltration contributes to the development of local and systemic inflammation [62]. IL-23, a cytokine derived from dendritic cells and macrophages, favors the development of CRC tumors [63]. On the other hand, fat is metabolized in adipocytes by different molecular pathways including peroxidation [64]. Metabolites generated from lipid peroxidation activate different signaling pathways, leading to various inflammatory responses or apoptosis [65]. Two of these products, 4-hydroxy-2-nonenal (4-HNE) and malondialdehyde (MDA), favor tumor development and progression by the effects they possess. Moreover, 4-HNE induces caspase-mediated apoptosis [66], forms covalent adducts on macromolecules such as proteins, DNA, and lipids, which, in mitochondria, modulate mitochondrial function and metabolic reprogramming [67], and inhibits DNA repair by inducing cyclooxygenase-2 (COX-2) and modulating the mitogen-activated protein kinase (MAPK), phosphatidylinositol 3-kinase (PI3K)/protein kinase B (PKB, best known as AKT), and protein kinase C (PKC) signaling pathways [68,69,70]. MDA has potent mutagenic effects [71] by inducing interstrand cross-linking and forms adducts upon reaction with proteins or DNA [72]. Thus, systemic inflammation and oxidative stress, in the form of lipid peroxidation, are the two key cellular and molecular processes in the relationship between obesity and the development of CRC (Figure 3).

Obesity may contribute to the inflammatory environment that supports the aggressive nature of CMS4 tumors as adipose tissues from obese individuals secrete proinflammatory cytokines that promote tumor progression and metastasis [34]. Furthermore, VAT is intimately linked to the tumor microenvironment (TME), where adipocytes contributing to tumor progression are located [61]. In this context, inflammasomes, large protein complexes of the innate immune system, promote tumor progression by facilitating inflammatory EMT through the activation of caspase-1 and consequent secretion of IL-1β and IL8 [73]. VAT increases NF-κB expression [74]. The constitutive activation of NF-κB promotes tumor development and progression [75], in part by regulating the chronic inflammatory processes [75]. Enlarged adipocytes in VAT shift their secretome to a proinflammatory profile [76]. The inflammatory environment, together with the adipocytes, results in the secretion of large amounts of adipokines and other cytokines that activate the transcription factors that promote tumor progression [77]. The macrophages and immune cells infiltrating VAT described above continue to release proinflammatory cytokines [62]. IL-6, secreted by macrophages and VAT-infiltrating B cells, could induce cancer proliferation through signal transducer and activator of transcription proteins (STAT) signaling [78], while TNF-α, released by macrophages and CD4+Th1 cells, would enhance mitogenic signaling through the c-Jun N-terminal kinases (JNK) pathway [79] (Figure 3).

Obesity is characterized by a permanent state of hyperleptinemia due to the secretion of leptin by adipose tissue. Elevated leptin expression, rare mutations, and single-nucleotide polymorphisms (SNPs) have been identified in individuals with moderate and severe obesity [80]. Moreover, individuals with moderate or severe obesity exhibit an elevated risk of mortality across all the stages of CRC. Therefore, a triangular relationship exists between leptin, obesity, and CRC [81].

The term “adiposopathy” refers to the dysfunction of adipocytes and adipose tissue that contributes to metabolic syndrome. This condition is characterized by adipocyte hypertrophy and their excessive abundance in various tissues [82]. Metabolic syndrome is defined as an accumulation of several disorders, including central obesity, insulin resistance, hypertension, and atherogenic dyslipidemia [83] (Figure 2). Metabolic syndrome is associated with an increased risk of CRC, CRC-specific mortality [84], and an increased risk of EOCRC [85]. Furthermore, metabolic dysfunction-associated steatotic liver disease (MASLD), which is the hepatic manifestation of adiposopathy and excessive fasting basal plasma glucose, is associated with CRC risk [86]. Insulin resistance is characterized by elevated plasma glucose concentrations and simultaneous hyperinsulinemia [87]. While insulin resistance does not precede weight gain, it is a consequence of weight gain [88]. Insulin resistance has been linked to the development of early-obesity-associated colorectal neoplasia [89]. Accordingly, C-peptide concentrations and insulin resistance are associated with the risk of developing CRA [87]. Insulin has been demonstrated to possess a direct tumorigenic effect through binding to the insulin receptor on target cells, as well as an indirect procarcinogenic effect by enhancing the synthesis and bioavailability of insulin-like growth factor 1 (IGF-1) [90]. Both circulating insulin and IGF-1 are increased in obesity [91]. However, the correlation between CRC risk and serum IGF-1 is relatively modest [92].

In conclusion, both metabolic syndrome and insulin resistance are linked to obesity-associated colon cancer through changes in insulin, and the IGF system [93,94]. Obesity and its associated metabolic consequences, such as insulin resistance and enhanced adipokine secretion, may contribute to the development and progression of CMS3 tumors. Indeed, metabolic syndrome is closely linked to this particular form of CRC [95].

An unhealthy diet in conjunction with a sedentary lifestyle represents the primary etiological factor underlying the development of obesity and CRC. On top of this association, diets high in red and processed meats, such as the Western diet [96], as well as diets high in fat and low in fiber and vitamins, augment the risk of CRC [97,98] (Figure 2). Red meat contains carcinogens including heterocyclic aromatic amines and polycyclic aromatic hydrocarbons that are formed during cooking at high temperatures [99,100]. Furthermore, meat processing (e.g., curing or smoking) can result in the formation of multiple carcinogens, including N-nitroso compounds [101]. Moreover, polyamines, which are found in processed red meat [102], have been associated with an elevated risk of CRC [103] through the APC-c-MYC pathway [104,105]. However, one particular polyamine, spermidine, may have CRC-preventive effects [106,107]. In addition, the increased risk of CRC associated with obesity has been attributed to an increase in circulating free fatty acids. There is a positive association between visceral obesity and the content of ω6 polyunsaturated fatty acids (PUFA) and an inverse association with monounsaturated fatty acids (MUFA) and ω3 PUFA in adipose tissue [108]. Ingested or diet-derived fatty acids may exhibit pro- or anti-inflammatory activity. For instance, long-chain saturated fatty acids (SFAs) and ω6 PUFAs may have proinflammatory effects, while short-chain fatty acids (SCFAs) derived from the microbial fermentation of nondigestible foods and ω3 PUFAs may have anti-inflammatory effects [108]. Elevated levels of SFAs in obesity have been linked to abnormal T-lymphocyte activation and increased Th17 cell responses, which promote colorectal tumor initiation and growth [109]. Patients with CRC exhibit an imbalanced PUFA ω3/ω6 ratio. This alteration is due to a significant decrease in ω3 PUFAs’ alpha-linolenic acid and stearidonic acid, along with an accumulation of ω6 PUFAs, such as dihomo-γ-linolenic acid and arachidonic acid, in the adipose tissue.

## 4. Molecular Factors Involved in the Development and Progression of Obesity-Associated Colorectal Cancer

In 2022, Hanahan redefined the hallmarks of cancer, identifying 14 key factors that build upon the previous 10 hallmarks. The new emerging hallmarks include non-mutational epigenetic reprogramming, polymorphic microbiomes, senescent cells, and unlocking phenotypic plasticity. The Decalogue of established hallmarks includes sustaining proliferative signaling, evading growth suppressors, preventing immune destruction, enabling replicative immortality, promoting tumor inflammation, activating invasion and metastasis, inducing angiogenesis, genome instability and mutation, resisting cell death, and deregulating cellular energetics [110]. The full catalogue of new and established hallmarks applies to CRC.

### 4.1. Genome Instability and Mutation and Non-Mutational Epigenetic Reprogramming

The initiation phase of CRC development involves genetic and epigenetic lesions resulting in CIN, MSI, and the CpG island methylation phenotype (CIMP) [111]. Up to 65–70% of sporadic CRCs present CIN, whose main features are the activation of WNT signaling, mutational inactivation of *APC*, and mutational inactivation/deletion of tumor protein 53 (*TP53*) [112], although mono-ADP ribosylhydrolase 2 (*MACROD2*) microdeletions can also be observed in a smaller fraction of this type of colorectal tumors [113]. In fact, *APC* inactivation is the most common mutation at adenoma onset because it is present in 80% of sporadic CRC [26]. However, mutations in Kirsten rat sarcoma virus (*KRAS*), loss of heterozygosity (LOH) at chromosome 18q, SMAD family member 4 (*SMAD4*), cell division control 4 (*CDC4*), and *TP53* promote CRC progression and metastasis [32,114].

**Figure 4:**
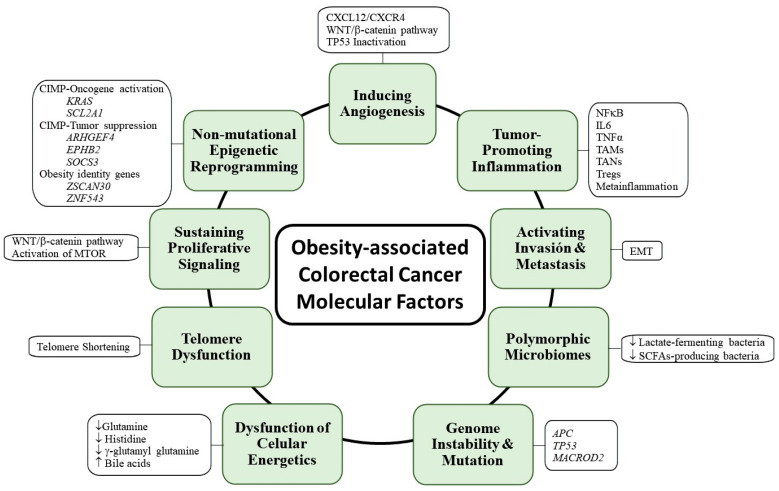
Obesity-associated colorectal cancer (CRC) molecular factors associated with cancer hallmarks. Schematic representation of major molecular targets involved in CRC related to obesity. APC: adenomatous polyposis coli; ARHGEF4: rho guanine nucleotide exchange factor 4; CIMP: CpG island methylation phenotype; CXCL12: C-X-C motif chemokine 12; CXCR4: C-X-C chemokine receptor type 4; EMT: epithelial–mesenchymal transition; EPHB2: EPH receptor B2; IL-6: interleukin 6; KRAS: Kirsten rat sarcoma virus; MACROD2: mono-ADP ribosylhydrolase 2; MTOR: mammalian target of rapamycin; NFκB: nuclear factor kappa-light-chain-enhancer of activated B cells; SCFA: short-chain fatty acid; SCL2A1: solute carrier family 2 member 1; SOCS3: suppressor of cytokine signaling 3; TAMs: tumor-associated macrophages; TANs: tumor-associated neutrophils; TNFα: tumor necrosis factor α; TP53: tumoral protein 53; Tregs: regulatory T cells; ZSCAN30: zinc finger and SCAN domain containing 30; ZNF543: zinc finger protein 543; ↑, high; ↓, low.

On the other hand, MSI results from MMR disruption. MSI occurs in approximately 15% of sporadic CRCs and is mostly associated with *MLH-1* hypermethylation [115,116], but also with phosphatase and tensin homolog (*PTEN*) hypermethylation in CRC tumors with high MSI (MSI-H) [117,118,119]. In addition, as mentioned above, Lynch syndrome results from germline mutations in DNA MMR genes, such as *MSH2*, *MLH1,* and less frequently *MSH6* and *PMS2* [29]. Associations between the methylation frequencies in the markers related to CIMP- and MSI-related markers confirm that MSI cancers arise from CIMP [120]. Genome-wide analysis studies in obese and CRC patients have identified hypermethylated CpG islands involved in oncogene activation, such as *KRAS* and solute carrier family 2 member 1 (*SCL2A1*), or tumor suppression, such as rho guanine nucleotide exchange factor 4 (*ARHGEF4*), EPH receptor 2 (*EPHB2*), and suppressor of cytokine signaling 3 (*SOCS3*), which may explain the cancer initiation in obese patients [121]. DNA methylation, abnormal distributions of differentially overlapping methylated regions such as hypermethylated CpG islands, contribute to the development of CRC in obesity. In addition, the altered DNA methylation of extracellular and intracellular components contributes to the activation of oncogenes and suppression of tumor suppressors, leading to increased oncogenic potency [121]. In addition, epigenetically regulated genes have been identified in CRC, including zinc finger and SCAN domain containing 30 (*ZSCAN30*, also known as *ZNF397OS*) and zinc finger protein 543 (*ZNF543*), which correlate with BMI and are able to discriminate obese from non-obese CRC patients [122] (Figure 4).

### 4.2. Enabling Replicative Immortality

Telomere dysfunction contributes to colorectal carcinogenesis [123]. Extensive telomere erosion occurs early in the development of CRA [124,125]. The SNPs of the genes involved in the telomere structure, maintenance or length have been associated with CRC risks, as documented for the protection of telomeres 1 (*POT1*) rs116895242, DNA cross-link repair 1B (*DCLRE1B*) rs12144215, telomerase RNA component (*TERC*) rs80304993 rs62293480 and rs75316749, telomerase reverse transcriptase *TERT* rs2736098, and *CTD-2194D22.4* rs12655062 [126]. Telomere dysfunction contributes to carcinogenesis through altering the stem cell dynamics. Thus, telomere dysfunction induces enhancer of zeste homolog 2 (EZH2) repression by APC-deficient cancer stem cells, resulting in the derepression of WNT antagonists, hence triggering the differentiation of the neighboring normal stem cells [127]. Of note, telomere shortening is associated with BMI in CRC patients, suggesting that obesity favors CRC pathogenesis through the erosion of telomeres [128]. In addition, telomere shortening in colonocytes is associated with the consumption of red (rather than white) meat and low dietary fiber intake [129] (Figure 4). Moreover, local tumor invasion is associated with telomere length (TL) in subcutaneous adipose tissue (SAT). Overweight CRC patients showed longer telomeres in both SAT and VAT [130].

### 4.3. Sustaining Proliferative Signaling

WNT/β-catenin is the main maintenance of proliferative signaling pathways in CRC [131]. In 2022, Zhao et al. published a comprehensive review on the involvement of the WNT/β-catenin signaling pathway in CRC. An abnormal WNT/β-catenin signaling pathway favors CRC development because it is involved in a complex network of protein–protein interactions influencing multiple biological processes, such as (1) cell proliferation, through *APC* mutations or the activation of KRAS/*B-Raf* proto-oncogene, serine/threonine kinase (BRAF)/MAPK signaling; (2) stemness, through hypoxia-inducible factor (HIF); (3) apoptosis of CRC cells by ectopic expression of the 2A-containing V-set and transmembrane domain (VSTM2A); (4) autophagy by upregulation of the a Na+/Cl-coupled neutral and cationic amino acid transporter SLC6A14 and inhibition of FAM134B (also known as the reticulophagy regulator 1, RETREG, or JK-1); (5) metabolism by upregulation of NADPH oxidases (NOX1), yielding hydrogen peroxide, which potentiates the WNT/β-catenin proliferation pathway; (6) chronic inflammation, through reactive oxygen species (ROS) production and increased expression of peroxisome proliferator-activated receptor γ (PPARγ); (7) microenvironment, through the B-cell lymphoma 9 (BCL9) oncogene, which promotes tumor progression and TME remodeling; (8) therapeutic resistance, involving guanylate-binding protein (GBP)-2 and miR 199a/b, among others; and (9) metastasis through the upregulation of various ion channels, such as chloride channel-coupled 1 (CLCA1), chlorine channel 3 (CLC-3), overexpression of the K channel potassium voltage-gated channel subfamily Q member 1 (KCNQ1), and transient receptor potential channel 5 (TrpC5) [132]. Epidermal growth factor receptor (EGFR)-MAPK is the other proliferative signaling pathway implicated in CRC. Increased *EGFR* expression is present in 25–77% of CRC cancers. EGFR activation induces the MAPK pathway, which includes *KRAS* and *BRAF*. *KRAS* is usually mutated in sporadic CRCs (35–45%), whereas *BRAF* mutations are found in approximately 5–10% of metastatic CRCs (mCRC) [114]. In addition to MAPK, the PI3K/AKT/mammalian target of rapamycin (MTOR) signaling pathway may be activated and involved in CRC development [133]. Obesity correlates with the activation of MTOR, which operates downstream of both the PI3K/AKT and MAPK pathways [134,135] (Figure 4).

### 4.4. Deregulating Cellular Energetics

Cancer cells overcome the growth constraints that govern normal cells by inactivating cell cycle checkpoints (through the upregulation of aurora kinase A and polo-like kinase 1, PLK1), tolerating DNA damage (primarily through deletion/degradation of TP53) and abrogating senescence (through the inactivation of TP53 and inhibition of cyclin-dependent kinase inhibitor 1, CDKN1A, best known as p21) [136]. The CMS3 subtype of CRC is enriched in KRAS mutant tumors with increased expression of the genes involved in various metabolic processes such as glucose, glutamine, glutathione, and lipid metabolism [34]. Metabolic reprogramming in CRC involves major glucose, lipid, and amino acid pathways. Some of the regulators of CRC metabolism are WNT, KRAS, TP53, MYC, and cystathionine-β-synthase (CBS) [137]. Most patients with advanced CRC (at least 25%) have liver metastases [138]. MCRC cells in the liver upregulate aldolase B, which promotes fructose metabolism, thereby increasing glycolysis and gluconeogenesis and promoting cell proliferation [139]. However, the reprogramming of lipid metabolism is what most affects the immune microenvironment in CRC as it is directly related to a high-fat diet that affects and alters the immune infiltrate [140]. Obesity-related metabolic alterations, such as metabolic syndrome, insulin resistance, altered lipid metabolism, endocrine changes, and oxidative stress, may promote CRC [70]. A metabolomic signature of BMI has been identified that is positively associated with CRC risk. Within this signature, glutamine, which has a cytoprotective effect, is inversely associated with CRC. Similarly, histidine and γ-glutamyl glutamine, which have anti-inflammatory effects, are inversely associated with CRC. Conversely, androsteroid monosulfate 2, a serum androgen metabolite, has been positively associated with CRC risk [141]. Obese patients also have elevated bile acids, which enhance the inflammatory processes and increase the damage to the intestinal epithelium, thereby increasing the risk of CRC [142] (Figure 4).

### 4.5. Promoting Tumor Inflammation

Systemic inflammation has been implicated in the pathogenesis of CRC. The characteristics of systemic inflammation include increased production of proinflammatory cytokines. This systemic inflammation promotes tumor growth, angiogenesis, and metastasis [143]. In obesity-associated CRC, NFκB is a critical regulator of inflammation through IL-6 production and TNFα activation [144,145]. In addition, the immunosuppressive TME is characterized by the presence of myeloid suppressor cells, such as tumor-associated macrophages (TAMs) and tumor-associated neutrophils (TANs), and regulatory T cells (Tregs) [146,147]. Thus, an obesity-related chronic low-grade inflammatory state, called “metainflammation”, may contribute to the failing immunosurveillance in CRC [108] (Figure 4).

### 4.6. Inducing Angiogenesis and Activating Invasion and Metastasis

Angiogenesis contributes to the invasion and dissemination of malignant cells, mainly through the vascular endothelial growth factor (VEGF) and its receptors (VEGFRs) [148]. The upregulation of VEGF initiates tumorigenesis by contributing to the activation of EMT [149]. EMT promotes the basement membrane invasion of cancer cells and ultimately cancer cell metastasis, including in CRC [150,151]. C-X-C motif chemokine 12 (CXCL12, also known as stromal cell-derived factor 1, SDF-1) and its receptor, C-X-C chemokine receptor type 4 (CXCR4), play an important role in angiogenesis and are associated with tumor progression. The WNT/β-catenin signaling pathway regulates obesity-associated CRC invasion and EMT induced by the activation of the CXCL12/CXCR4 axis [152,153,154]. Finally, inactivation of TP53 increases intestinal permeability, initiating NFκB-dependent inflammation and the induction of EMT [155].

### 4.7. Polymorphic Microbiomes

Obesity induces gut microbiota dysfunction, and gut microbiota dysfunction is associated with EOCRC [156]. In patients with ulcerative colitis and Crohn’s disease, intestinal dysbiosis has been associated with the development of CRC [157]. Microbial dysbiosis may lead to the secretion of inflammatory mediators such as TNF-α, ILs, and IFNs, thus favoring mutations in stem cells and progressive dysplasia of the colon epithelium [158]. Certain bacteria can interact with the tumor through oncometabolites that promote cancer progression [159]. Alterations in the fecal and mucosal microbiota with reduced ecological diversity have been reported in CRC patients. CRC patients show an increase in Bacteroidetes and decrease in Firmicutes, especially of the Clostridia class, which ferment dietary fiber and other carbohydrates to butyrate, an SCFA that reduces colonic inflammation and carcinogenesis. Eleven microbial species have been identified as direct human carcinogens (oncomicrobes), such as, for example, some strains of *Escherichia coli* that produce colibactin, a potent DNA alkylator associated with CRC [160]. Increased abundances of the *Fusobacterium*, *Atopobium*, and *Porphyromonas* genera are also associated with CRC [161]. The other microbial taxa found in CRC patients include *Porphyromonas*, *Peptoniphilus*, *Fenollaria*, *Finegoldia*, *Ezakiella*, *P. urinae*, *F. massiliensis*, *A. vaginalis*, *F. magna*, *E. coagulans*, *Str. salivarius*, *P. faecalis*, and *P. asaccharolytica* [162]. The genera *Porphyromonadaceae*, *Lachnospiraceae* UCG010, *Lachnospira*, and *Sellimonas* have a positive association with CRC risk, while the *Lachnospiraceae* species have a negative association with CRC risk [163]. *E. coli*, *Enterococcus faecalis*, *Bacteroides fragilis*, *Streptococcus bovis*, and *Peptostreptococcus anaerobius* are implicated in CRC initiation, whereas *Bacteroides fragilis* is associated with CRC promotion due to the toxins it produces and *Fusobacterium* with CRC progression. In sharp contrast, *Akkermansia muciniphila* and *Faecalibacterium prausnitzii* may prevent CRC [164,165,166]. Among the genera found to be increased in obese individuals is *Fusobacterium* [167], which is also abundant in CRC. Furthermore, the abundance of SCFA-producing bacteria decreases in both obese and CRC patients [168]. These changes could be a cause or consequence, so the relationship between dysbiosis in obese patients and its impact on CRC development remains difficult to establish.

Diet modulates the intestinal microbiota. Thus, a high-protein diet reduces the production of beneficial SCFAs in the gut, while a high-fat diet increases the pathogenic microbes in the gut, reduces lactate-fermenting and SCFA-producing bacteria, and increases the production of lysophosphatidic acid and deoxycholic bile acid. Conversely, a low-fat diet causes the enrichment of *Bifidobacterium*, a beneficial genus of bacteria. Dietary fibers are carbohydrates that are metabolized by colonic microbiota and whose products are SCFAs, such as butyrate. Dietary fiber intake facilitates the proliferation of fiber-degrading bacteria, such as *Lactobacillus* and *Bifidobacterium* [164]. Deoxycholic acid and lithocholic acid are secondary bile acids produced by the colonic microbiota from the primary bile acids cholic acid and chenodeoxycholic acid, which are produced by the liver from cholesterol metabolism. Secondary bile acids, in turn, can modulate the composition of the intestinal microbiota and modulate tumor development [169,170]. Thus, deoxycholic acid causes the non-canonical activation of the EGFR/MAPK pathway in colon cancer cells [171]. Because bile secretion is increased in a high-fat diet, regular consumption of this type of diet may promote tumor development secondary to shifts in the gut microbiota and increased local inflammation (Figure 4).

## 5. Current Treatments of Obesity-Related Colorectal Cancer: Perspective and Challenges

Currently, there are no diagnostic or monitoring methods for the early detection of CRC in patients with obesity, and the same procedures are followed as for normal-weight individuals [172]. Given the increase in obesity in young people and the association between obesity and CRC risk, several United States task forces, including the American Cancer Society and the US Preventive Services Task Force, support CRC screening in people younger than 50 years. Obesity-related cancers account for nearly 43.5% of the total direct costs associated with cancer care in the US [173]. In addition, obesity increases the risk of surgical site infection after colectomy by 60%, and the presence of infection increases the cost of care [174]. Therefore, the implementation of preventive strategies, both policy and clinical, is needed. A US nutrition policy study associated the labeling of added sugars in product nutrition information with a reduction in new cancer cases and cancer deaths, as well as savings in the medical costs associated with cancer care in adults across the lifespan [175]. Yeoh et al. calculated the potential clinical impact and cost-effectiveness of earlier (40 years) or more intensive (colonoscopy every 10 to 5 years or annual fecal immunochemical test (FIT)) CRC screening in obese individuals (I–III). Based on the results obtained, CRC screening starting at age 45 years with a colonoscopy or at age 40 years with FIT appears to be cost-effective for women and men across the BMI range. However, a colonoscopy every 10 years from age 40 years appears to be cost-effective only for men with a BMI of II–III, even though this population has the highest risk of CRC [176].

Similarly, there are no specific treatments for obesity-associated CRC. Although there have been significant changes in the treatment of CRC over the past 30 years, the efficacy of these therapies is highly variable. Recently, some important therapeutic innovations have been implemented, as exemplified by (1) the use of magnetic resonance imaging (MRI) and optimized computed tomography (CT) scanning for diagnosis and follow-up [177]; (2) total mesorectal excision, which consists of removing the tumor en bloc with its blood and lymphatic supply, reducing local recurrence [178]; and (3) treatment with radiotherapy and chemotherapy before surgery [177]. However, obese patients present several challenges. These include technical challenges due to the weight and opening limitations of the CT/MR table, as well as difficulties in performing endoscopy and obtaining endoscopic biopsies [179].

The main treatment for CRC is surgery. The data on the correlation between obesity and the surgical outcomes related to CRC indicate that there is no association between BMI and short-term mortality, long-term mortality, or major surgical complications. However, there is an association between BMI and an increased incidence of minor surgical complications, such as wound dehiscence and infection [180]. Radiotherapy or chemotherapy may be used before and/or after surgery. The protocol depends on the stage of CRC. CRC is divided into five different stages. Patients with clinical stages I–IIA colon cancer are treated with surgery. In stages III, IV (advanced), and V (metastatic), patients undergo adjuvant chemotherapy prior to surgery. The recommended chemotherapy is a combination regimen of fluorouracil (5-FU)/leucovorin and oxaliplatin for 6 months or capecitabine and oxaliplatin for 3 months [181,182]. If a patient is ineligible for oxaliplatin, capecitabine or 5-FU/leucovorin may be employed as an alternative [181]. To date, there is no difference between obese and non-obese CRC patients. Optimal doses of individual or combined chemotherapeutic treatments are calculated based on body surface area in adult patients. Therefore, a priori, obese patients should not experience greater toxicity than healthy-weight individuals. However, it should be noted that up to 40% of obese patients may be at risk of being undertreated [179]. This may be because obese patients have an increased risk of peripheral neuropathy, which limits the dose. Alternatively, physicians may calculate the dose based on ideal body weight or an arbitrary dose limit to avoid toxicity in these patients. In either case, undertreatment is associated with increased relapse and mortality in obese patients [183]. Therefore, alternative anthropometric measures such as waist-to-hip ratio or body composition, which may result in different treatment distributions in patients of the same body weight, and physiological variables that determine drug clearance should be considered [183].

### 5.1. Surgery and Radiotherapy

Surgery varies depending on the type and size of the neoplasm [184]. For CRAs, cold- or hot-loop polypectomy can be performed [185], as well as endoscopic submucosal dissection (ESD) for polyps larger than 2 cm [186] and early-stage CRC [187]. Local resection is a procedure used to minimize invasiveness and reduction in bowel function. It is recommended in the early stages and for some high-risk CRCs [188]. Men and obese patients have a narrow pelvis and middle and lower rectal tumors, which limits laparoscopic total mesorectal excision for rectal cancer in these patients. For these cases, transanal total mesorectal excision is a new technique designed to overcome these limitations [189]. If the cancer is too large, a bowel resection is performed to remove the cancer and surrounding healthy tissue [190]. In some cases, surgery is not possible or inefficient. Most trials report no difference in surgery between obese and lean patients, and the overall evidence is inconclusive [7]. Transperitoneal approaches can be technically challenging in obese patients, so an extraperitoneal approach for supine left colon resections is currently being evaluated, with promising results [191]. In addition, surgery in obese patients presents anesthesia-related challenges, such as high-risk airways and associated diagnosed or undiagnosed comorbidities, such as diabetes mellitus, cardiovascular disease, and obstructive sleep apnea. In addition, patients with obesity are at high risk for postoperative complications [179]. Laparoscopic surgery is increasingly used for the treatment of CRC. However, obese patients have one of the highest overall conversion rates to open surgery due to technical difficulties [192,193]. Robotic surgery appears to be a better option in obese patients. However, obesity in these patients is associated with a longer duration of robotic colorectal surgery and an increased risk of wound infection [194]. Radiation therapy employs high-energy X-rays or other forms of radiation to eradicate the cancer cells or stop their proliferation [195]. In certain instances, intraoperative radiation therapy is employed [196].

### 5.2. Chemotherapy

#### 5.2.1. Fluoropyrimidines

Chemotherapy can be administered in a systemic or regional manner. The classical chemotherapy agents for CRC include fluoropyrimidines, irinotecan, and oxaliplatin. Fluoropyrimidines encompass 5-FU/leucovorin, capecitabine, tegafur/uracil, and trifluridine/tipiracil. Given that the majority of CRC patients are elderly (over 70 years of age), the management of these patients presents additional challenges associated with their age, such as other comorbidities, functional status, cognitive function, and frailty [197].

All the treatments are summarized in Table 1.

The combination of 5-FU with leucovorin represents a first-line treatment for CRC. Indeed, 5-FU, a thymidylate synthase inhibitor, is incorporated into the nucleic acids of tumor cells, resulting in differential cytotoxicity [198,226]. Leucovorin, or folinic acid, is a vitamin that enhances the efficacy of 5-FU while reducing the adverse effects [198,226]. Similarly to 5-FU, capecitabine is a first-line treatment for CRC [199]. Capecitabine is a prodrug that is converted to 5-FU by thymidine phosphorylase. The advantage of capecitabine over 5-FU is that thymidine phosphorylase is highly expressed in cancer cells, resulting in a markedly enhanced cytotoxic effect in tumor cells [199]. Tegafur is another 5-FU precursor that is used as a first-line therapeutic agent for the treatment of CRC. This prodrug is metabolized to 5-FU by cytochrome P450 family 2 subfamily A member 6 (CYP2A6). Tegafur is administered with uracil to prevent the action of dihydropyrimidine dehydrogenase on 5-FU, thereby increasing its bioavailability [200,201]. The combination of trifluridine and tipiracil is employed in patients who have already undergone treatment with oxaliplatin-based fluoropyrimidines or irinotecan, or in patients who are unable to receive these types of treatments. In this context, trifluridine/tipiracil represents a third- or fourth-line treatment for mCRC [202]. Trifluridine is a thymidine-based nucleoside analog that can be phosphorylated by thymidine kinase and incorporated into DNA, resulting in the formation of single- and double-strand breaks [203]. Trifluridine is rapidly metabolized by thymidine phosphorylase. For this reason, trifluridine is administered in conjunction with tipiracil, a potent inhibitor of thymidine phosphorylase [203].

#### 5.2.2. Topoisomerase I Inhibitors

Irinotecan is a prodrug that is metabolized to ethyl-10-hydroxy-camptothecin (SN38). SN38 is a topoisomerase I inhibitor that produces DNA breaks, triggering DNA damage checkpoint signaling (ataxia-telangiectasia mutated serine/threonine kinase (ATM)- checkpoint kinase 2 serine/threonine kinase (CHK2)-TP53) and thus leading to apoptosis [204]. Deruxtecan is another topoisomerase I inhibitor that has demonstrated efficacy in combination with targeted therapy of mCRC [205].

#### 5.2.3. Platinum-Based Drugs

Oxaliplatin is a third-generation platinum-derived anticancer drug that is also utilized as a first-line treatment for CRC. Oxaliplatin forms adducts with cellular DNA, thereby altering key processes such as DNA replication and transcription [206,207,214].

### 5.3. Combined Therapies

In clinical practice, a combination of multiple agents from the same or different pharmacological groups are employed to treat CRC. For example, there are several established combination regimens that are utilized as the initial treatment options. FOLFOX is a combination of leucovorin, oxaliplatin, and 5-FU. FOLFIRI is a combination of leucovorin, 5-FU, and irinotecan. FOLFOXIRI is a combination of leucovorin, 5-FU, oxaliplatin, and irinotecan [221]. These combinations represent the fundamental structure of the treatment, and different combinations with targeted therapy are being tested with positive outcomes [222,223,224]. Combinations with radiotherapy are also a common occurrence [225].

### 5.4. Targeted Therapy

In addition to the classical chemotherapeutic treatments, targeted therapy can be an invaluable tool to halt CRC progression as more than 50% of CRC patients present a very specific molecular profile [208].

#### 5.4.1. VEGF Inhibitors

Given that the VEGF/VEGFR axis is involved in CRC progression and metastasis [209], the monoclonal antibody directed against VEGF-A, bevacizumab, is employed in first- and second-line strategies for mCRC [210], with a significant impact on overall survival [211]. Obesity reduces the efficacy of bevacizumab treatment against CRC [7]. Aflibercept (VEGF trap), a recombinant fusion protein comprising VEGFR-1 and -2 domains that functions as a “trap” sequestering VEGF-A and -B, is not recommended as a first-line therapy due to increased adverse effects. A similar conclusion can be drawn about ramucirumab, an immunoglobulin (Ig)G antibody that targets VEGFR-2 [211]. Agents that interfere with VEGF signaling have been associated with significant adverse effects on the cardiovascular and gastrointestinal systems, as well as hematologic effects. Consequently, they are not typically recommended as first-line therapy [212].

#### 5.4.2. BRAF Inhibitors

As previously stated, CRC can manifest in two forms: wild-type or mutated BRAF. The mutations can be either BRAF V600E or BRAF non-V600E. The former has a dismal prognosis, a worse response to chemotherapy, as well as a higher incidence of peritoneal metastases [215]. Vemurafenib, dabrafenib, and encorafenib are BRAF inhibitors with comparable potency. In a phase III clinical trial, the combination of the three BRAF inhibitors increased the overall survival and objective response rate in BRAF V600E-mutated mCRC compared to the standard therapy [216].

#### 5.4.3. EGFR Inhibitors

At last, two monoclonal antibodies, cetuximab and panitumumab, which are directed against EGFR, are employed in the initial or subsequent treatment of CRC, in combination with chemotherapy against KRAS wild-type CRC [217,218].

#### 5.4.4. Immune Checkpoint Inhibitors

The potential of immunotherapy against CRC has recently been investigated. Immune checkpoint inhibitors targeting cytotoxic T-lymphocyte-associated antigen 4 (CTLA-4), programmed death 1 (PD-1), and its ligand (PD-L1) are increasingly used in oncological practice [220]. This therapeutic approach has demonstrated efficacy in a select group of CRC patients with MSI-H [219]. Among these therapies we find novolumab and pembrozozumab, monoclonal antibodies directed against PD-1; atezolizumab and durvalumab, monoclonal antibodies specific to PD-L1; and ipilimumab and tremelimumab, monoclonal antibodies directed against CTLA-4 [220]. A favorable outcome has been documented in patients with advanced CRC who received combination therapy with immune checkpoint inhibitors and regorafenib, a multikinase inhibitor that targets VEGF, platelet-derived growth factor (PDGF), and tyrosine kinases [213]. An important trend in the field concerns the preoperative (neoadjuvant) administration of immune checkpoint inhibitors. Thus, it appears that neoadjuvant immunotherapy (mostly with PD-1 or PD-L1 blocking antibodies, alone or in combination with CLTA-4 blockade) and chemoimmunotherapy (a combination of oxaliplatin-based chemotherapy and immunotherapy) will become the future standard of care for CRC even when the tumor lesions are operable. There are multiple examples suggesting that this kind of approach can lead to complete pathological responses that avoid the surgical resection of the cancer [227,228,229,230].

Given that obesity is a risk factor for CRC, interventions that result in weight loss may have a dual effect, both as a preventive measure and as a therapeutic intervention. For instance, a reduction in CRC mortality and an improved prognosis have been observed in patients with moderate to high levels of physical activity [231].

## 6. New Targets for Obesity-Associated Colorectal Cancer

The guidelines by the European, American, and Canadian health authorities recommend CRC patients to adopt lifestyle modifications, including healthy diets, increased physical activity, stress reduction, and the establishment of optimal sleep habits. Psychological interventions may be recommended to achieve these changes [232,233,234]. In instances where these recommendations prove insufficient or when the patient’s health is significantly compromised, the use of obesity medications and bariatric surgery may be indicated [232,233,234,235]. Of note, bariatric surgery exhibits a 35% reduction in the risk of developing CRC compared with obese individuals who had no surgery [236].

In addition to anthropometric measurements indicative of overweight and obesity, selected parameters reflecting metabolic syndrome (hyperglycemia, hyperinsulinemia, hypertriglyceridemia, hypercholesterinemia, high LDL and low high-density lipoprotein (HDL), and hypertension) and inflammation (high C-reactive protein and IGF-1) can elevate the risk of CRC [237,238,239,240]. Hyperglycemia has been demonstrated to trigger several mechanisms that may be involved in the development of CRC [241] or even favor hepatic metastasis of CRC [242]. In contrast, hypertriglyceridemia has not been consistently associated with an increased risk of CRC in most of the studies; the sample size of these studies was small [239]. It is noteworthy that hepatosteatosis resulting from obesity provides an optimal environment for CRC liver metastasis [243,244,245]. In contrast, the evidence for an association between hypertriglyceridemia and an increased risk of CRC is inconclusive, with inconsistent findings across studies [239]. However, a fivefold increased risk of CRC was observed in a Taiwanese population study comparing diabetic patients to non-diabetic patients. Within the diabetic cohort, the risk of CRC was fourfold higher in male patients with elevated triglycerides compared to those without [246]. This finding has been corroborated by another study from Korea that identified a correlation between persistent hypertriglyceridemia and EOCRC [247].

### 6.1. Thiazolidinediones

In addition, in a prediabetes state, elevated plasma insulin levels may be found. Insulin promotes growth and influences IGF-1 levels [239]. A US analysis found a causal effect of fasting insulin levels on the development of CRC [248]. A number of pharmacological agents are currently employed for the management of the blood glucose levels in individuals with diabetes and prediabetes. Thiazolidinediones (TZDs), such as troglitazone, rosiglitazone, and pioglitazone, which act as PPARγ agonists, are employed to enhance the peripheral insulin sensitivity in individuals with diabetes and prediabetes. A meta-analysis of diabetic patients revealed that treatment with TZDs resulted in a 9% reduction in the risk of developing CRC [249]. Given that PPARγ has been demonstrated to exert anti-inflammatory effects [250], the impact of TZDs may be twofold, namely the reduction in glucose levels and the attenuation of inflammation.

It has recently been described that activation of PPARγ by pioglitazone and rosiglitazone significantly increases the sensitivity of CRC cell lines to 5-FU [251], as occurs with rosiglitazone and 5-FU in hepatocellular carcinoma cell lines [251]. On the other hand, nanoparticles loaded with capecitabine and pioglitazone appear to be an effective strategy against CRC [252]. The use of TZD in combination with chemotherapy has also been studied in a preclinical rat model of breast cancer [253]. However, other previous studies had observed no effect of rosiglitazone plus oxaliplatin in human colon cancer cells [254].

### 6.2. Metformin

Another drug utilized for the management of glucose levels is metformin. The use of this drug in diabetic patients has been associated with a reduced incidence of CRC in various population groups [255,256]. Metformin has been shown to selectively inhibit *KRAS*-mutated mCRC [257]. Multiple studies have examined the efficacy of combination therapies involving metformin in CRC, for example, the combination of doxorubicin, metformin, and sodium oxamate in vitro [258], the combination therapy of sorafenib and metformin in vivo [259], or metformin + 5-FU/oxaliplatin (FuOx) in vivo and in vitro [260]. In fact, following metformin administration increases chemosensitivity to oxaliplatin [261,262]. Similarly, the encapsulation of metformin with amphiphilic liposomes constructed with oxaliplatin prodrugs effectively potentiates immune checkpoint blockade (ICB) therapy against murine colorectal tumors [263]. Moreover, preclinical studies have shown that the combination of metformin with PD-1 blockade potentiates PD-1 blockade [264]. However, the administration of nivolumab and metformin has not demonstrated efficacy on tumor progression in patients with stable microsatellite mCRC in a phase II clinical trial (NCT03800602) [265]. Finally, metformin also does not reduce the number or size of colorectal polyps in patients with FAP (NCT01725490) [266].

### 6.3. Sulfonylureas

It was unexpected to find that the use of another traditional glycemic control medication, sulfonylurea, was associated with an increased risk of CRC in diabetic patients aged 65 and above. However, among the sulfonylureas, gliclazide was observed to protect against the development of CRC. In the same study, no effect of other antidiabetic drugs, including TZDs and metformin, on CRC risk was reported [267].

### 6.4. DPPA Inhibitors and the GLP-1 Mimetic Semaglutide

Dipeptidyl peptidase IV inhibitors (DPP4i), such as sitagliptin, enhance the bioavailability of incretins (glucagon-like peptide 1, GLP-1, and glucose-dependent insulinotropic peptide, GIP). These incretins stimulate β-pancreatic cells by increasing insulin release and reducing the postprandial and fasting blood glucose levels [268]. The use of DPP4i has been associated with a reduced CRC risk [269]. GLP-1 receptor (GLP-1R) agonists are long-lived GLP-1 mimetics that stimulate insulin release, inhibit glucagon production, and protect pancreatic beta cells. This class of drugs has recently gained attention due to the approval of semaglutide by the Food and Drug Administration (FDA) and European Medicines Agency (EMA) for the treatment of both type 2 diabetes and obesity. This drug has demonstrated efficacy in the long-term reduction of body weight [270]. These effects were observed in both sexes and across all the ethnicities studied, although one of the inclusion criteria was a BMI ≥ 27 kg/m^2^ [271]. A recent retrospective study demonstrated a decreased risk of CRC in the GLP-1R agonist-treated group compared with the insulin-treated group, but not compared with the metformin-treated group [272]. Conversely, another study demonstrated an elevated risk of CRC in patients undergoing semaglutide [273].

To our knowledge, the efficacy of DPP4i alone or in combination with chemotherapy has not been evaluated to date in studies with CRC, although there are studies in other types of cancer with promising results, such as in prostate cancer patients [274], in combination with paclitaxel in ovarian cancer in vitro and in vivo [275,276]. Consequently, further research is required to elucidate the impact of this category of pharmaceuticals on CRC incidence.

### 6.5. Alpha-Glucosidase Inhibitor

Conversely, α-glucosidase inhibitors diminish carbohydrate absorption in the small intestine by inhibiting several enzymes, including glucoamylase, sucrase, maltase, and isomaltase. Some studies indicate a reduction in the incidence of CRC with this drug [277,278].

### 6.6. SGLT2 Inhibitors

Sodium–glucose transporter-2 inhibitors (SGLT2i), such as canagliflozin and dapagliflozin, are pharmaceutical agents that are specifically formulated to regulate blood glucose levels. SGLT2 is expressed in the proximal tubule of the kidney and functions to reabsorb up to 80% of the glucose filtered in the nephron. Consequently, the inhibition of this process results in the excretion of a considerable quantity of glucose, thereby enhancing glycemic control [279]. The currently available data present conflicting results regarding the association between SGLT2i, particularly empagliflozin, and cancer. Notwithstanding the aforementioned controversy, the improvement in the diabetic CRC patients treated with SGLT2i appears to be a clear outcome. Nonetheless, SGLT2i can be used to efficiently treat diabetes in patients with CRC [280,281,282,283]. The preclinical experimentation suggests that SGLT2i has antineoplastic effects on CRC cells [284,285,286,287].

SGLT2is have also been evaluated in recent years as enhancers of the efficacy of chemotherapy and/or radiotherapy [283]. Thus, it has been observed that canagliflozin synergizes the effect of radiotherapy and docetaxel in prostate and lung cancer cells [288], of doxorubicin in liver and breast cancer cells [289], and of radiotherapy in non-small-cell lung cancer (NSCLC) [290], although it does not affect the efficacy of cisplatin in lung and colon cancer cells [291]. For its part, dapagliflozin improves the response to paclitaxel in breast cancer in vitro and in vivo [292,293]. Clinical trials are currently underway to evaluate the clinical efficacy of combining SGLT2i with chemotherapy in pancreatic carcinoma (NCT05903703), metastatic breast cancer with PIK3CA mutation (NCT05090358), and locally advanced and/or metastatic pancreatic ductal adenocarcinoma (PDAC) (NCT04542291). The authors are unaware of similar studies in CRC.

### 6.7. Statins

Dyslipidemia is a common finding in obese patients, occurring in approximately 60–70% of individuals with a BMI greater than 30 kg/m^2^ and in 50–60% of those with a BMI between 30 and 25 [82]. As previously stated, hypertriglyceridemia is linked to an elevated risk of CRC [246,247]. Another common disorder associated with obesity is lipoprotein imbalance, which is characterized by low levels of HDL or high levels of very-low-density lipoprotein (VLDL) and small, dense LDL [82]. The evidence regarding the contribution of elevated cholesterol levels to CRC risk is inconclusive, with some studies reporting an increased risk and others reporting no effect [294,295]. Nevertheless, these alterations in lipid levels have been demonstrated to activate proinflammatory pathways [296]. Statins are a class of pharmaceutical agents that are prescribed for the purpose of reducing cholesterol levels. The use of statins for a period of at least ten years does not increase the risk of developing cancer, including CRC. Indeed, there is evidence that statins may have an antitumor effect in a range of cancers, including CRC [297,298]. Furthermore, a recent study has demonstrated that the short-term administration of statins (90 days) prior to surgery in patients with stages I–III CRC has the potential to enhance the overall survival rates [299].

Statins have demonstrated a synergistic effect combined with chemotherapy in *CRC* in vitro and in vivo. Lovastatin has been observed to increase the efficacy of 5-FU or cisplatin in vitro [300] and of irinotecan in vivo [301]. In addition, the use of a liposomal carrier co-loaded with SN38, the active metabolite of irinotecan, and lovastatin (SL@Lip) has been shown to significantly potentiate both chemotherapy and immunotherapy (αPD-1) [302]. Simvastatin and fluvastatin have demonstrated efficacy as adjuvants to oxaliplatin in *KRAS*-mutated CRC [303], while regorafenib and rosuvastatin combination therapy has shown synergistic effects both in vitro and in vivo [304]. The efficacy of statins combined with chemotherapy has also been studied with positive results in other types of cancer. For example, ruthenium–fluvastatin [305], cloxuridine and camptothecin nanocapsules loaded with lovastatin [306] and pentoxifylline plus simvastatin [307], atorvastatin plus doxorubicin [308], or simvastatin combined with 5-FU, adriamycin, and cyclophosphamide (FAC) [309] have been studied in different types of breast cancer. Other studies include atorvastatin plus carboplatin in NSCLC [310], lovastatin plus doxorubicin in ovarian cancer cells [311], lovastatin plus paclitaxel in prostate cancer cells [312], lovastatin plus [177Lu]Lu-trastuzumab-based radioligand therapy in drug-resistant gastric cancers [313], atorvastatin plus gemcitabine in human cholangiocarcinoma cells [314], simvastatin plus temozolomide in glioblastoma cells [315], or simvastatin plus doxorubicin in neuroblastoma cells [316]. However, the phase III clinical trials to date have not demonstrated the superior efficacy of statin plus chemotherapy combinations over chemotherapy alone in either CRC (NCT01238094) [317] or advanced gastric cancer (NCT01099085) [318], or in small-cell lung cancer (NCT00433498) [319], or in advanced hepatocellular carcinoma (NCT01075555) [320]. In light of these findings, statins should be explored for combination with chemotherapy in the treatment of patients with obesity-associated CRC.

### 6.8. Fenofibrates

Conversely, fenofibrate, a PPARα agonist utilized for the reduction in triglyceride levels, has demonstrated encouraging outcomes in vitro and in animal models [321]. Fenofibrate has anti-inflammatory activity, suggesting that the antitumor effect may result not only from an improvement in the metabolic profile but also from a reduction in inflammation.

Fenofibrate sensitizes docetaxel- and mitoxantrone-resistant prostate cancer cells [322]. However, to our knowledge, there are no further data on the efficacy of the combined use of fenofibrate with chemotherapy in any type of cancer.

### 6.9. Leptin Inhibitors

Adipose tissue has been demonstrated to possess endocrine functions, as indicated by its capacity to produce adipokines and steroids such as glucocorticoids or sex hormones [323]. In individuals with obesity, adipose tissue releases elevated levels of leptin. A positive correlation between leptin levels and CRC risk has been demonstrated [324,325,326]. Therefore, both adipokines represent potential therapeutic targets for CRC in obese patients. Leptin is associated with CRC not only due to elevated levels but also as a result of genetic variants. The presence of different leptin polymorphisms may result in varying degrees of CRC risk [327,328].

As with CRC, obesity is also a risk factor for pancreatic cancer. The in vivo treatment with iron oxide nanoparticle-leptin peptide receptor antagonist 2 (IONP-LPrA2) delays the onset and decreases the tumor growth [329]. Furthermore, it appears that IONP-LPrA2 could improve 5-FU therapy in patients with pancreatic cancer, especially if they have obesity [330], as could the combination of IONP-LPrA2 with paclitaxel in type II endometrial cancer [331], or IONP-LPrA2 with cisplatin, cyclophosphamide, and doxorubicin in breast cancer, in particular for triple negative breast cancer (TNBC) [332]. The use of leptin inhibitors or leptin receptor antagonists should be explored in the context of CRC, particularly in cases where leptin genetic variants are present and confer an elevated risk.

### 6.10. Rapalogues and PI3K/AKT Inhibitors

Leptin and insulin/IGF-1 activate MTOR complex 1 (MTORC1) through the activation of the MAPK or PI3K pathways. Increased MTOR expression has been associated with CRC [333]. Indeed, MTORC1 activation occurs in individuals with obesity [135], suggesting that the inhibition of MTORC1 with rapamycin (sirolimus) or rapalogues (everolimus, ridaforolimus, and temsirolimus) may be considered as a strategy for CRC prevention. Nevertheless, when inhibiting MTORC1, one must consider the potential compensatory activation of the PI3K/AKT pathway, which would increase the risk of cancer. A combined inhibition of MTORC1 and PI3K/AKT may prove to be the optimal approach [135].

A pilot study shows that the combined regimen of 5-FU, irinotecan, bevacizumab, and sirolimus, after the failure of classical therapy is promising for advanced CRC [334]. Other therapeutic combinations with MTORC1 and PI3K inhibitors together with chemo- or radiotherapy have been tested with some success in other cancers: (1) docetaxel or 5-FU plus temsirolimus in human prostate cancer cells and human breast cancer xenografts [335]; (2) MTOR inhibitors with therapies in patients with hormone-receptor-positive metastatic breast cancer (mBC-HR+) [336], especially effective in those patients with alterations in the PI3K/AKT/mTOR pathway [337]; (3) everolimus combined with an aromatase inhibitor in patients with mBC-HR+ (NCT00863655) [338]; (4) everolimus plus endocrine therapy in postmenopausal women with aromatase-inhibitor-resistant MBC (NCT01805271), although with problems with tolerability of everolimus [339]; (5) carboplatin with sirolimus or everolimus in canine melanoma cell lines [340]; (6) everolimus plus cisplatin in lung cancer cells in vitro [341] and in urothelial bladder cancer [342]; (7) temsirolimus potentiates the activity of gemcitabine and cisplatin in bladder cancer [343]; (8) combination of sirolimus, PD-L1 antibody, and gemcitabine in a murine model of PDAC [344]; (9) everolimus plus cyclophosphamide in gastric cancer in vivo [345,346]; and (10) everolimus plus vinorelbine in renal cell carcinoma [347]. Finally, dual PI3K-mTOR inhibitors have also shown efficacy both alone and in combination. For example, BEZ235 has been shown to be effective in pancreatic cancer xenografts [348], in anaplastic thyroid cancers when combined with paclitaxel [349], and in lung cancer alone or combined with cisplatin [350], as well as PF-04979064 combined with 5-FU for gastric cancer [351].

### 6.11. ACBP/DBI Blocks

Finally, we would like to discuss a phylogenetically ancestral hormonal factor, diazepam-binding protein (DBI), which is also known as acyl-CoA-binding protein (ACBP). ACBP/DBI is expressed and released from human or mouse cells in response to autophagy induction, making it an actionable target that can be neutralized by monoclonal antibodies [352,353,354]. It is noteworthy that elevated ACBP/DBI expression is linked to obesity and metabolic syndrome in mice and humans [355,356]. Indeed, obese patients who have undergone bariatric surgery exhibit a reduction in the plasma levels of ACBP, which correlates with the weight loss achieved [357]. Of note, in mice, the neutralization of ACBP/DBI reduces appetite and protects against obesity and metabolic dysfunction-associated steatohepatitis (MASH, formerly known as nonalcoholic steatohepatitis or NASH) induced by various diets [357,358,359,360]. Several studies indicate the potential involvement of ACBP/DBI in carcinogenesis and tumor progression [355]. Elevated expression of ACPB/DBI is observed in specimens from patients with glioma and glioblastoma. Knockdown of ACPB/DBI reduces the proliferation of glioblastoma cells, likely due to a reduction in fatty acid oxidation [361,362]. Additionally, ACBP/DBI has been involved in the pathogenesis of other tumor types, including bladder cancer [363], breast cancer [364], non-small-cell lung cancer [365], cholangiocarcinoma [366], and hepatocellular carcinoma [367]. In human CRC, ACBP/DBI is overexpressed relative to the adjacent normal tissue [368]. Similarly, the ACBP expression is elevated in transformed rat colon epithelial cells [369]. In light of these findings, it can be proposed, yet remains to be demonstrated, that ACBP/DBI constitute a multipronged target for simultaneous interventions on obesity, metabolic syndrome, and CRC.

To our knowledge, no studies combining ACBP/DBI, α-glucosidase inhibitors, semaglutide, empagliflozin, or sulfonylureas with traditional chemotherapy have been performed to date to evaluate their efficacy.

### 6.12. Pre-Pro Biotics/FMT

Probiotics include lactic acid bacteria, such as *Lactobacillus* and *Bifidobacterium*, but also *Streptococcus*, *Pediococcus*, *Leuconostoc*, *Enterococcus,* and the yeast *Saccharomyces boulardii* [370,371]. The new-generation probiotics include *Prevotella copri* and *Christensenella minuta*, which control insulin resistance; *Parabacteroides goldsteinii*, *Akkermansia muciniphila*, and *Bacteroides thetaiotaomicron*, which reverse obesity and insulin resistance; *F. prausnitzii*, which protects mice against intestinal diseases; and *Bacteroides fragilis*, which reduces inflammation and shows anticancer effects [372]. *Pediococcus pentosaceus* K28, *Levilactobacillus brevis* RP21, and *Lactiplantibacillus plantarum* RP12, isolated from grains, can inhibit adipogenesis in adipocytes, characterized by reduced lipid accumulation and decreased expression of adipogenic markers in vitro. Therefore, they could also be useful as probiotics [373]. The combined administration of *Lactobacillus rhamnosus* LC705 and *Propionibacterium freudenreichii ssp shermanii* JS in healthy men has decreased the activity of β-glucosidase, a bacterial enzyme that contributes to the development of CRC by producing carcinogens [374].

Prebiotics are nondigestible dietary ingredients that stimulate the growth of the beneficial bacteria of the intestinal microbiota [375]. The effects of prebiotics, such as galactooligosaccharides (GalOSs) and the combination of GalOS and inulin, have been evaluated in a colon cancer model induced with 1,2-dimethylhydrazine dihydrochloride. GalOS increases populations of beneficial bacteria (Bifidobacteria and Lactobacilli) and decreases concentrations of harmful bacteria, reduces the formation of aberrant crypt foci, and shows an increased level of SCFA (acetate, propionate, and butyrate) [376,377]. On the other hand, yogurt consumption, compared to no or little consumption, has been associated with a lower probability of adenomas [378,379,380]. Similarly, higher yogurt consumption has been associated with a lower relative risk of incident CRC compared to no yogurt consumption [381,382].

The combination of probiotics and prebiotics (synbiotics) has also shown efficacy against the development of CRC. In an azoxymethane (AOM)-induced rat model of CRC, male Sprague-Dawley rats fed a combined prebiotic and probiotic diet consisting of a moderate intake of resistant starch in combination with *Bifidobacterium lactis* showed greater protection against CRC than those fed such a diet without B. lactis [383,384]. The combination of the prebiotic oligofructose-enriched inulin and the probiotics *Bifidobacterium lactis* and *Lactobacillus rhamnosus* protects rats against AOM-induced colon cancer [385]. Some data in patients show that polypectomized colon cancer patients subjected to a symbiotic preparation of oligofructose-enriched inulin (SYN1) + *Lactobacillus rhamnosus* GG (LGG) and *Bifidobacterium lactis* Bb12 (BB12) showed an increase in *Bifidobacterium* and *Lactobacillus*, as well as a decrease in *Clostridium perfringens*. This intervention reduced the proliferative capacity of colonocytes, decreased the necrosis of these same cells, and improved the epithelial barrier function in polypectomized patients, reducing the risk of CRC in these patients [386]. On the other hand, dietary supplementation with oligosaccharides or fiber has increased the levels of short-chain fatty acid-producing bacteria, thus inhibiting tumorigenesis. In addition, as adjuvants to surgery or chemotherapy, Lactobacilli and Bifidobacteria decrease complications [387].

To our knowledge, there are no studies in which such interventions have been performed in in vivo models or in obese patients with CRC.

On the other hand, chemotherapy can result in dysbiosis [388]. As dysbiosis is associated with CRC, interventions on the microbiota might reduce the risk of CRC and serve as potential therapeutic targets. Indeed, nanoparticle delivery of prebiotics enhances the efficacy of chemotherapy in CRC in mice [389]. While there are numerous methods for influencing microbial composition, including the use of pre- and/or probiotics, fecal microbiota transplantation (FMT) represents the most direct approach for manipulating the bacterial composition in the context of CRC. However, FMT is not exempt from severe adverse effects, particularly in immunosuppressed patients [164]. Many studies focus on the alteration that chemotherapy causes in the intestinal microbiota and how the intestinal microbiota modulates the efficacy of these treatments. However, there are hardly any studies that focus on how to improve the efficacy of the traditional treatments by modifying the intestinal microbiota. A recent study in patients with gastric cancer shows that *Akkermansia muciniphila* and its metabolite pentadecanoic acid may support the efficacy of oxaliplatin, suggesting the possibility of probiotic and prebiotic interventions in conjunction with chemotherapy [390].

### 6.13. Epigenetic Therapies

Epigenetic aberrations are associated with drug resistance [391]. Epigenetic drugs such as DNA methylation inhibitors (DNMTis) and histone deacetylase inhibitors (HDACis) are approved in monotherapy for cancer treatment. In addition, these drugs act synergistically with other epigenetic drugs or with various CRC drugs [392]. DNMTi-based epigenetic therapy, such as 5-azacitidine (5-AC) or 5-aza-2′-deoxycytidine (decitabine, DAC), improves the sensitivity to both irinotecan and 5-FU [393]. In addition, the combination of DAC with 5-FU or oxaliplatin improves the treatment of patients with CRC [394]. On the other hand, DNMTi causes increased neoantigen presentation by MHC class I on tumor cells and leads to increased neoantigen-specific T-cell activation in combination with radiotherapy in patients with MSS-RCC [395]. DNMTi potentiates the effect of radiotherapy and immunotherapy by demethylating the PD-L1 promoter and increasing radiotherapy-induced PD-L1 upregulation via IFN-β in vivo [396]. Combination treatment of low-concentration regorafenib, a multiple kinase inhibitor, and the small-molecule Janus kinases (JAK)/ histone deacetylases (HDAC) dual JAK/HDACi inhibitors (JAK/HDACi), to potentiate antitumor activity by inhibiting both JAK and HDAC, shows superior efficacy to these drugs alone in the treatment of mCRC. The randomized phase II CAPability-01 trial (NCT04724239) determined that the combination of PD-1 antibody with the HDACis chidamide and bevacizumab in patients with unresectable locally advanced or mCRC could be a promising treatment regimen for patients with advanced CRC [397]. HDACi pracinostat suppresses CRC by inducing peripheral-mitophysis-mediated CDK5- dynamin-related protein 1 (Drp1) signaling [398]. To our knowledge, there are no studies in which such interventions have been performed in in vivo models or in obese CRC patients.

Table 2 summarizes the potential new therapeutic targets for obesity-associated CRC.

## 7. Impact of Dietary Compounds on the Prevention and Treatment of Obesity-Associated Colorectal Cancer

Various bioactive nutraceutical compounds present in foods have been identified as beneficial for the prevention of cancer.

Dietary ω3 PUFAs, including alpha-linolenic acid (ALA, 18:3n-3), docosapentaenoic acid (DHA, 22:5), and eicosapentaenoic acid (EPA, 20:5n-3), and their endocannabinoid mediators, are associated with obesity and the development of CRC [399]. Dietary ω3 PUFAs, mainly DHA and EPA, attenuate adipose tissue inflammation in several animal models of obesity [400]. Therefore, DHA and EPA may prevent the progression of obesity [401]. In fact, ω3 PUFAs are involved in the reduction in cytokines such as IL-1, IL-6, and TNF-α, whose levels are elevated in obesity [402]. The epidemiological evidence indicates that diets rich in ω3 PUFAs or dietary supplementation with ω3 PUFAs may offer a protective effect against CRC. A recent study has demonstrated that fish consumption and dietary intake of ω3 PUFA are associated with a reduced CRC risk [108]. Accordingly, the Mediterranean diet, which is rich in vegetables and fruit and PUFAs contained in olive oil and fish, but poor in SFAs, reduces the risk of developing obesity and CRC [403,404,405]. Indeed, the regular consumption of a Mediterranean diet has been shown to reduce the risk of CRC by approximately 21% [406]. Furthermore, the efficacy of ω3 PUFA in suppressing CRC development has been demonstrated in a carcinogen-induced, diabetes-promoted animal model and confirmed in vitro. Therefore, ω3 PUFA would have prospective therapeutic potential in the treatment of CRC, especially that promoted by diabetes [407]. For prevention, perhaps the minimum EPA+DHA intake for healthy adults should be the minimum that has been shown to reduce obesity, i.e., 0.5–1 g/day (2–4 weekly servings of fish, half of them oily fish) [408], corresponding to a total ω3 PUFA ration of 5–6 g/day [409].

Polyphenols, such as curcumin, resveratrol, epigallocatechin, and quercetin, are molecules with antioxidant, anti-proliferative, anticancer, and anti-obesity properties.

Curcumin (diferuloylmethane), a polyphenol found in turmeric, reverses the symptoms associated with obesity-related cancers, such as insulin resistance, suggesting that curcumin could be considered in the treatment and prevention of obesity-related cancers [410]. Curcumin may suppress proinflammatory cytokines as well as inflammatory pathways [411,412] and prevent colon cancer [413]. A study in an in vivo model of obesity-associated colon cancer using high fat diet (HFD)-fed A/J mice treated with AOM shows that the combination of curcumin with salsalate, a non-steroidal anti-inflammatory drug (NSAID), reduces colonic cytokines (IL-1β, IL-6, and TNF-α) and the activation of the PI3K/AKT/MTOR and NF-κB pathways in the colonic mucosa [414,415], reduces the tumor burden by 80% [415], and also reduces aberrant crypt foci in humans [416]. However, curcumin has low bioavailability, which limits its clinical use. Therefore, the use of curcumin derivatives is currently being evaluated. The data to date suggest that they are potential therapeutic candidates with the ability to modulate obesity and obesity-related complications [417].

Resveratrol, a natural polyphenolic compound found mainly in grape skins and seeds, suppresses colon cancer cell proliferation in vitro by inhibiting the IGF-1 receptor (IGF-1R)/AKT/WNT signaling pathways and p53 activation [418], induces apoptosis through the pentose phosphate and talin-FAK-A signaling pathways [419], decreases the nuclear localization of β-catenin-attenuated WNT/β-catenin signaling, and reduces the expression of target genes c-Myc and MMP-7, leading to the inhibition of CRC invasion and metastasis [420].

Epigallocatechin, or (-)-epigallocatechin-3-gallate (EGCG), is the major polyphenolic compound found in green tea and has anti-obesity and anti-diabetic effects. In a study conducted in an in vivo model of obesity-related colon cancer, CRC was induced by AOM in C57BL/KsJ-db/db/db (db/db) mice that are obese and develop diabetes mellitus, EGCG was observed to decrease the levels of IGF-1R, phosphorylated form of IGF-1R (p-IGF-1R), p-GSK-3β, β-catenin, COX-2, cyclin D1, insulin, triglycerides, cholesterol, and leptin, as well as the development of premalignant lesions. This means that EGCG reduces the activation of the IGF/IGF-1R axis, inhibits obesity-related events such as hyperlipidemia, hyperinsulinemia, and hyperleptinemia, and reduces the incidence of CRC [421]. EGCG modulates lipid metabolism by disrupting lipid emulsification and absorption, suppressing adipogenesis and lipid synthesis, and increasing energy expenditure through thermogenesis, fat oxidation, and fecal lipid excretion [422]. In addition, the combination of EGCG with sodium butyrate, a product of the intestinal microbiota, promotes apoptosis, induces cell cycle arrest, and damages the DNA of CRC cells in vitro [423,424].

Quercetin, a flavonoid found in fruits and vegetables, is able to reduce the carcinogenesis in male C57BL/ksJ-db/db mice treated with AOM [425]. The combination of quercetin with probiotics such as *Bifidobacterium bifidum* and *Lactobacillus gasseri* inhibits the CRC development in *ApcMin*/+ mice by inhibiting the canonical WNT/β-catenin pathway [426].

Therefore, polyphenols may be useful in the chemoprevention or treatment of obesity-related CRC, although more research is needed to confirm these data as the existing data regarding patients are scarce. A study conducted among black individuals in the US showed that a higher polyphenol intake was associated with a lower risk of CRC or rectal cancer, and the association was consistent according to the BMI [427].

## 8. Concluding Remarks

Obesity represents a pathology affecting more than 890 million adults globally. CRC is one of the many diseases that can be considered as a comorbidity of obesity. At present, there are more than two million individuals afflicted with CRC on the global scale. CRC is a multifactorial disease in which certain risk factors, including obesity, increase the number of cases. Despite the decline in the mortality rates associated with CRC, as a result of the public screening campaigns implemented in various countries, the incidence of CRC developing in individuals under the age of 50 has been increasing. This phenomenon is likely attributable to lifestyle factors. An unhealthy diet, including diets high in processed food and red or processed meat, in conjunction with a sedentary lifestyle, represents the primary etiological factor underlying the development of obesity and CRC. Furthermore, individuals with moderate or severe obesity exhibit an elevated risk of mortality in patients with stages I–III CRC. Special attention should be paid to obese patients with Lynch syndrome, in whom the risk of CRC is doubled. In addition, to date, there are no diagnostic or follow-up methods for the early detection of CRC in patients with obesity, although lowering the age in screening programs would favor patients with obesity. The precise mechanism by which obesity promotes tumor onset and/or progression remains unclear. It is established that obesity is associated with a state of chronic inflammation in the patient, characterized by the accumulation of M1-polarized macrophages, Tregs, and the secretion of proinflammatory factors, including TNF-α, IL-6, IL-1β, and leptin, which have all been linked to CRC. Conversely, metabolic syndrome and insulin resistance are linked to obesity-related CRC through alterations in insulin and the IGF system. Systemic inflammation has been demonstrated to promote tumor growth, angiogenesis, and metastasis. Furthermore, obesity-associated CRC exhibits epigenetic alterations, telomere shortening, and the activation of trophic pathways. Furthermore, alterations in the intestinal microbiota and the subsequent elevation of secondary bile acids contribute to an enhanced inflammatory state, a reduction in the abundance of beneficial bacteria, and an increase in the prevalence of pathogenic bacteria. This creates an environment conducive to inflammation and tumor progression. Despite the advancements in the treatment of CRC over the past three decades, there remains a lack of targeted therapies for obesity-associated cancer. However, it should be noted that some of the current treatments, such as bevacizumab, have demonstrated reduced efficacy in obese individuals, perhaps because the patients are at risk of being undertreated. Furthermore, interventions resulting in weight loss, the use of certain anti-obesity medications, bariatric surgery, and adequate physical activity levels are associated with a reduction in CRC mortality. Bariatric surgery as a CRC preventive strategy should be weighed against the health benefits and risks associated with surgery. There is also evidence of success combining obesity-related drugs and the traditional treatments in other types of cancer, which should be explored in CRC. Given the established link between obesity and CRC, as well as the interference of obesity with certain treatments and the efficacy of combining anti-obesity drugs with the traditional treatments in other types of cancer, it is imperative to explore these new therapeutic strategies aimed at CRC patients with obesity or metabolic syndrome. It appears highly plausible that the reversal of obesity will have a major positive impact on the prognosis of CRC patients. To reduce the risk of CRC in the population, it is important to be aware of the importance of a healthy lifestyle and to practice it. Community education and awareness efforts would be critical in this regard. However, more clinical trials are needed before lifestyle modification in combination with drugs can be proposed as a treatment.

## Figures and Tables

**Table 1:** Current, in-development, and under-study therapies in colorectal cancer.

Treatment		Techniques/Drugs	Stages	References
Surgical resection	Polypectomy	Forceps or snare	CRA	[185]
	ESD	-	CRA > 2 cm and early-stage	[186,187]
	Local Excision	-	Early-stages	[188]
	Colon Resection	Colectomy- anastomosis	III–V	[190]
		Colostomy		
Radiation	External (X-rays)	-	Various stages	[195]
	Intraoperative			[196]
Chemotherapy	Fluoropyrimidines	5-FU/Leucovorin	First line, III–IV (II high-risk patients)	[198]
		Capecitabine	First line, III–IV	[199,200,201]
		Tegafur/Uracil	Third or fourth line, III–IV	[202,203]
		Trifluridine/Tipiracil	Third or fourth line, III–IV	[202,203]
	Topoisomerase I Inhibitors	Irinotecan	III–V	[204]
		Deruxtecan	III–V	[205]
	Platinum-based drugs	Oxaliplatin	First line, III–V	[206,207]
Targeted Therapy	VEGF Inhibitors	Bevacizumab	First and second line, IV–V	[7,208,209,210,211]
		Aflibercept	Second line, IV–V	[211,212]
		Ramucirumab	Second line, IV–V	[211,212]
		Regorafenib	IV–V	[213,214]
	BRAF Inhibitors	Vemurafenib/Dabrafenib/Encorafenib	IV–V	[215,216]
	EGFR Inhibitors	Cetuximab/Panitumumab	First or second line	[217,218]
	Immune Checkpoint Inhibitors	Nivolumab, Pembrozizumab, Iplizumab, Tremelimumab, Atezolizumab, Durvalumab	IV–V	[219,220]
Combined Therapies	-	FOLFOX	Different stages	[221]
		FOLFIRI		[222,223]
		FOLFOXIRI		[224,225]

5-FU: fluorouracil; ESD: endoscopic submucosal dissection; FOLFIRI: leucovorin/5-FU/irinotecan; FOLFOX: leucovorin/oxaliplatin/5-FU; FOLFOXIRI: leucovorin/5-FU/oxaliplatin, irinotecan; VEGF: vascular endothelial growth factor; VEGFR: VEGF receptor.

**Table 2:** New possible therapeutic targets for obesity-associated colorectal cancer.

Treatment	Actionable Target	Disease-Associated CRC	References
ACBP/DBI blocks	Anorexigenic activation/lipid synthesis inhibition	Metabolic syndrome	[352,353,354]
α-glucosidase inhibitor	Chemical digestion of glucide inhibition	T2D	[277,278]
DPP4i	Incretins availability	T2D	[268]
Empagliflozin	SGLT2 inhibition	T2D	[279,280,281,282,284,285,287]
Epigenetic inhibitors	DNMTi and HDACi	T2D and obesity	[392]
Leptin inhibitor	Appetite inhibition/energy metabolism	Obesity	[326]
Metformin	AMPK and SIRT1 activator	T2D and obesity	[256,287]
Pre-pro biotics/FMT	Alteration in microbiota composition	Metabolic syndrome	[164,389]
Rapalogues	mTOR inhibition	Obesity	[135]
Semaglutide	GLP-1 receptor agonist	T2D and obesity	[270,271]
Statins	HMG-CoA reductase inhibitor	Hypercholesteronemia/Dyslipidemia	[297,298,299]
Sulfonylureas	Insulin release	T2D	[267]
TZDs	PPARγ agonist	T2D	[250,271]

ACBP: Acyl–CoA-binding protein; AMPK: AMP-activated protein kinase; DBI: diazepam-binding protein; DPP4i: dipeptidyl peptidase IV inhibitors; DNMTi: DNA methylation inhibitors; FMT: fecal microbiota transplantation; GLP-1: glucagon-like peptide-1; HDACi: histone deacetylase inhibitors; HMG-CoA: 3-hydroxy-3-methyl-glutaryl-coenzyme A; mTOR: mammalian target of rapamycin; PPARγ: peroxisome proliferator-activated receptor gamma; SGLT2: sodium–glucose transport protein 2; SIRT1: Sirtuin 1; T2D: type 2 diabetes. TZCs: thiazolidinediones.
